# THREE DECADES OF PERSON-CENTERED DEMENTIA CARE: LESSONS LEARNED AND FUTURE CONSIDERATIONS

**DOI:** 10.1093/geroni/igae098.1840

**Published:** 2024-12-31

**Authors:** Sam Fazio

**Affiliations:** Alzheimer’s Association, Chicago, Illinois, United States

## Abstract

Dr. Fazio has been involved in person-centered dementia care for more than three decades. In this presentation, Dr. Fazio will review the origins of person-centered care and ongoing research, as well as share highlights of his personal research and his dedicated work at the Alzheimer’s Association. At the same time, he will discuss how we might need to reconsider the overall concept and its implementation for greater impact.

